# LIVIVO – the Vertical Search Engine for Life Sciences

**DOI:** 10.1007/s13222-016-0245-2

**Published:** 2017-01-18

**Authors:** Bernd Müller, Christoph Poley, Jana Pössel, Alexandra Hagelstein, Thomas Gübitz

**Affiliations:** Applied Research and Innovation, German National Library of Medicine ZB MED – Information Centre for Life Sciences, Gleueler Str. 60, 50931 Cologne, North Rhine-Westphalia Germany

**Keywords:** Literature Search, Knowledge Discovery, LIVIVO, Life Sciences, Information Retrieval, Text Mining, Data Mining

## Abstract

The explosive growth of literature and data in the life sciences challenges researchers to keep track of current advancements in their disciplines. Novel approaches in the life science like the One Health paradigm require integrated methodologies in order to link and connect heterogeneous information from databases and literature resources. Current publications in the life sciences are increasingly characterized by the employment of trans-disciplinary methodologies comprising molecular and cell biology, genetics, genomic, epigenomic, transcriptional and proteomic high throughput technologies with data from humans, plants, and animals. The literature search engine LIVIVO empowers retrieval functionality by incorporating various literature resources from medicine, health, environment, agriculture and nutrition. LIVIVO is developed in-house by ZB MED – Information Centre for Life Sciences. It provides a user-friendly and usability-tested search interface with a corpus of 55 Million citations derived from 50 databases. Standardized application programming interfaces are available for data export and high throughput retrieval. The search functions allow for semantic retrieval with filtering options based on life science entities. The service oriented architecture of LIVIVO uses four different implementation layers to deliver search services. A Knowledge Environment is developed by ZB MED to deal with the heterogeneity of data as an integrative approach to model, store, and link semantic concepts within literature resources and databases. Future work will focus on the exploitation of life science ontologies and on the employment of NLP technologies in order to improve query expansion, filters in faceted search, and concept based relevancy rankings in LIVIVO.

## Interdisciplinary Research and Vertical Information Search

### Why We Need Interdisciplinary Research and Development

Diseases and their treatment are one of the greatest challenges of our time. Infectious diseases, especially by multi-resistant pathogens, are a major and massively increasing cause of mortality [10]. The complexity of health issues and interactions between animal and human health have been shown by global epidemics like *Ebola*, *SARS*, or *H1N1*. The novel paradigm of *One Health* provides a global and trans-disciplinary approach to tackle infectious diseases at the human, animal, and environmental interface [3]. In order to follow the integrated approach of One Health, a wide variety of information sources from all life science disciplines need to be considered in research [1].

### Challenges of Interdisciplinary Literature Search and the Vertical Search Engine LIVIVO

Literature search and review is a time-consuming task that is often cut short by focusing on only a few, well known sources, like specific journals or databases such as PubMed, Google Scholar, Scopus, or Web of Science. The databases Scopus and Web of Science are usually fee-based being hardly affordable for students and researchers, especially in developing countries. As a results, many researchers are forced to rely on the few databases licensed by their institution or library, or they need to rely on free but limited sources such as PubMed and Google Scholar, restricting their literature search results to only a fraction of the relevant literature available.

ZB MED – Information Centre for Life Sciences is a non-profit organization with the mission to supply specialized literature in the life sciences at a national and an international level. ZB MED hosts the largest stock of life science literature in Europe while offering specialized search portals for literature search in life sciences. Since 2015, the most comprehensive search portal for life sciences LIVIVO^1^ offers the possibility for interdisciplinary literature search.

LIVIVO covers in depth the areas of medicine and health as well as nutritional, environmental and agricultural sciences, enabling users to exploit the synergy effects of interdisciplinary searches. More than 55 million literature references from about 50 national and international databases build the corpus of LIVIVO. This includes Medline (PubMed^2^), Agricola^3^, AGRIS, the catalog of the National Library of Medicine^4^, and other resources.

LIVIVO supports the Open Access Initiative by integrating open access literature from relevant databases such as German Medical Science^5^, the Electronic Journals Library (EZB) [6], and a selection of life science publications from the Bielefeld Academic Search Engine BASE [11].

LIVIVO’s usability-tested user interface [12] is easy and intuitive to use with any mobile device due to responsive design and is available in English and German. In order to increase the precision of search queries, results are automatically analyzed and enhanced with descriptors providing semantic structure for fine-tuning the query. For this purpose, three multilingual life science thesauri are used (MeSH, UMTHES and AGROVOC). These are also used for subject indexing of the meta-data listed in LIVIVO, enhancing the content-based indexing of the original data. Index terms can later be used in the portal for refining the precision of the search. For example, a query for the German term *Antibiotika-Resistenz* is connected to the thesauri automatically finding documents with the terms *Antimicrobial drug resistance* and *antibiotic / antimicrobial resistance*. Thus by expanding search terms, the semantic search technology of LIVIVO is effectively widening the search and increasing recall.

Open research data found in repositories on the web is automatically linked via digital object identifiers (DOI) to the citation in LIVIVO. This unique feature can be further used for filtering for publications enriched with research data. Personal relevance rating and additional content-related insight can be provided on the basis of abstracts, descriptions, keywords and table of contents, which can be displayed in the view of the search result.

## LIVIVO’s Service Oriented Architecture Provides Semantic Content

The underlying 55 million bibliographic meta records form the base of LIVIVO’s search services. They are collected from more than 50 data sources using heterogeneous original data formats, e.g.the relatively detailed Marc21^6^, MAB^7^, Medline^8^, the rather compact Dublin Core^9^, or proprietary (XML-)formats. In addition, the meta-data has different levels of quality regarding the information content.

The meta-data format used for LIVIVO’s search services is characterized by a relatively flat data structure. Its main purpose is to give simple and fast access for information retrieval with the Solr^10^-based search engine. Furthermore, the format provides the ability to store additional information such as semantic-linguistic annotations as well as the accessibility of documents.

LIVIVO includes meta-data converters closing the gap between the original format of the data providers and the retrieval-optimized LIVIVO data format. In some cases information loss and pruning can occur when information is transcribed into the LIVIVO data format. Therefore mappings of original data sources needs to be optimised in order to avoid loss of relevant information.

The meta-data converters also include an automated enrichment and linking of these data among themselves and with added information originating from different sources. In addition, the data converters perform their tasks automatically and thus guarantee the continuous update of the LIVIVO data index. The data converters are used to automatically update the LIVIVO index. The update frequency is either daily or weekly, dependant on the data sources.

There is an essential need to build an architecture with well defined interfaces and reusable components in order to provide user driven services based on modern software development. The architecture of LIVIVO is designed to achieve service oriented development by introducing four layers as technical solution that deliver search services. These four layers are:

- the web interface,
- the portal software,
- a query standardization layer, and
- the base layer of the search engine.

**Fig. 1:**
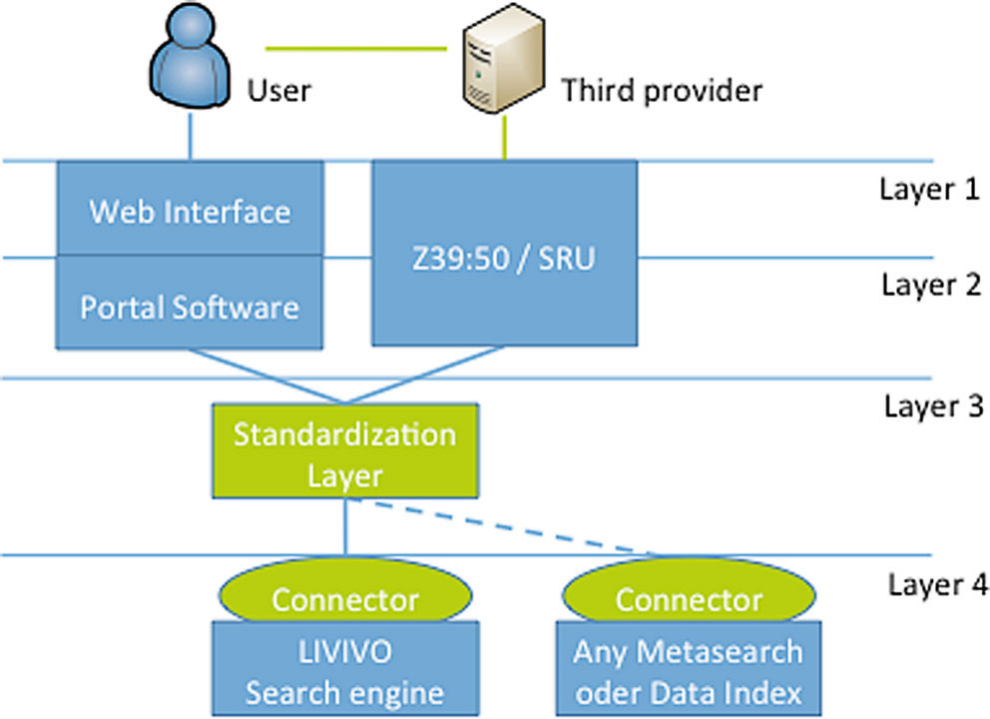
Service oriented architecture of LIVIVO with the four layers for the web interface, the portal software, the query standardization, and the base layer of the search engine

The general architecture with the 4 layers is visualized in Fig. 1.

### The Web Interface and Portal Software of LIVIVO

The Web Interface defines the access point for the user’s communication with LIVIVO. The interface features a conjunction to the portal software while using open source frameworks such as the Solr search platform. The task of the portal software is to process all user activities and to interact with the underlying layers of the LIVIVO architecture. Additionally, the portal software of LIVIVO is linked to a number of other web services such as the ZB MED customer management and the ZB MED document delivery. There is also a system that determines the availability of documents in the Electronic Journals Library (EZB).

In order to provide query decomposition, a standardization layer is developed that normalizes any query in LIVIVO for further processing. The standardization includes meaningful simplifications and completions of search terms and Boolean operators.

The standardized search queries can be transformed for further processes depending on the target format of the consuming service. Currently, LIVIVO uses a connector to the Solr-based search engine and data index. The connector first conducts the final search query. Then, data preparations such as weighting search elements are employed. Important factors for weighting search results are as follows:

- *Date*: The publication date of the citation
- *Occurrence*: A higher weight is given for search terms occurring in the title or the author names
- *Exact Matches*: If search terms are matched exactly to occurrences in the metadata, they will get a weighting boost

The following examples describes the query decomposition in conjunction with Boolean operators of the query

zoonoses (virus or bacteria)

In the standardization layer, the query is decomposed into the search terms:

- *zoonoses*
- *virus*
- *bacteria*

Then, the Boolean operators are assigned according to the parenthesis in the search query. This results in the Boolean query

zoonoses AND (virus OR bacteria)

Finally, boosting factors are assigned to the search fields combined with the decomposed Boolean query. Exemplary, the resulting query is extended for the two fields TITLE and AUTHOR with bf being the respective boosting factor:

$$\begin{aligned}&\displaystyle\text{TITLE:(zoonoses AND (virus OR bacteria))}{}^{\text{bf1}}\,\,\,\,{\displaystyle\text{OR}}\\ &\displaystyle\text{AUTHOR:(zoonoses AND (virus OR bacteria))}{}^{\text{bf2}}\end{aligned}$$

In the service oriented architecture, plug-ins provide a straightforward solution to implement features in LIVIVO. For example, there is a plug-in that enriches search results with language-independent concepts and phrases that are also stored in the LIVIVO data index. Another plug-in conducts the automated classification of documents into subjects like Medicine, Health, Nutrition, Environment and Agriculture. A similar plug-in is also used by the German National Library for an automated subject indexing of the growing number of electronic publications [15].

### Using LIVIVO as a Provider for Search Services of Third Parties

The general use case for LIVIVO is to provide a searchable virtual library on the web. In several cases, costumers are interested in using the search service of LIVIVO as a data driven service without any functionality of the web portal. Therefore, a high-performance, standardized and automated interface is available as encapsulated service, namely a Z39:50- and SRU interface. This encapsulated service is docked on the same layer as the web interface and the portal software in the service oriented architecture. This data-driven service features almost the complete functionality for searching and filtering queries as the web portal. The option to make use of LIVIVO’s search capacities without using the web portal creates novel user scenarios. Examples of the novel user scenarios are the integration of LIVIVO as a discovery service into existing portal solutions. Another example is the integration into literature management systems such as EndNote^11^, RefWorks^12^, and Citavi^13^.

## The ZB MED Knowledge Environment (KE)

**Fig. 2:**
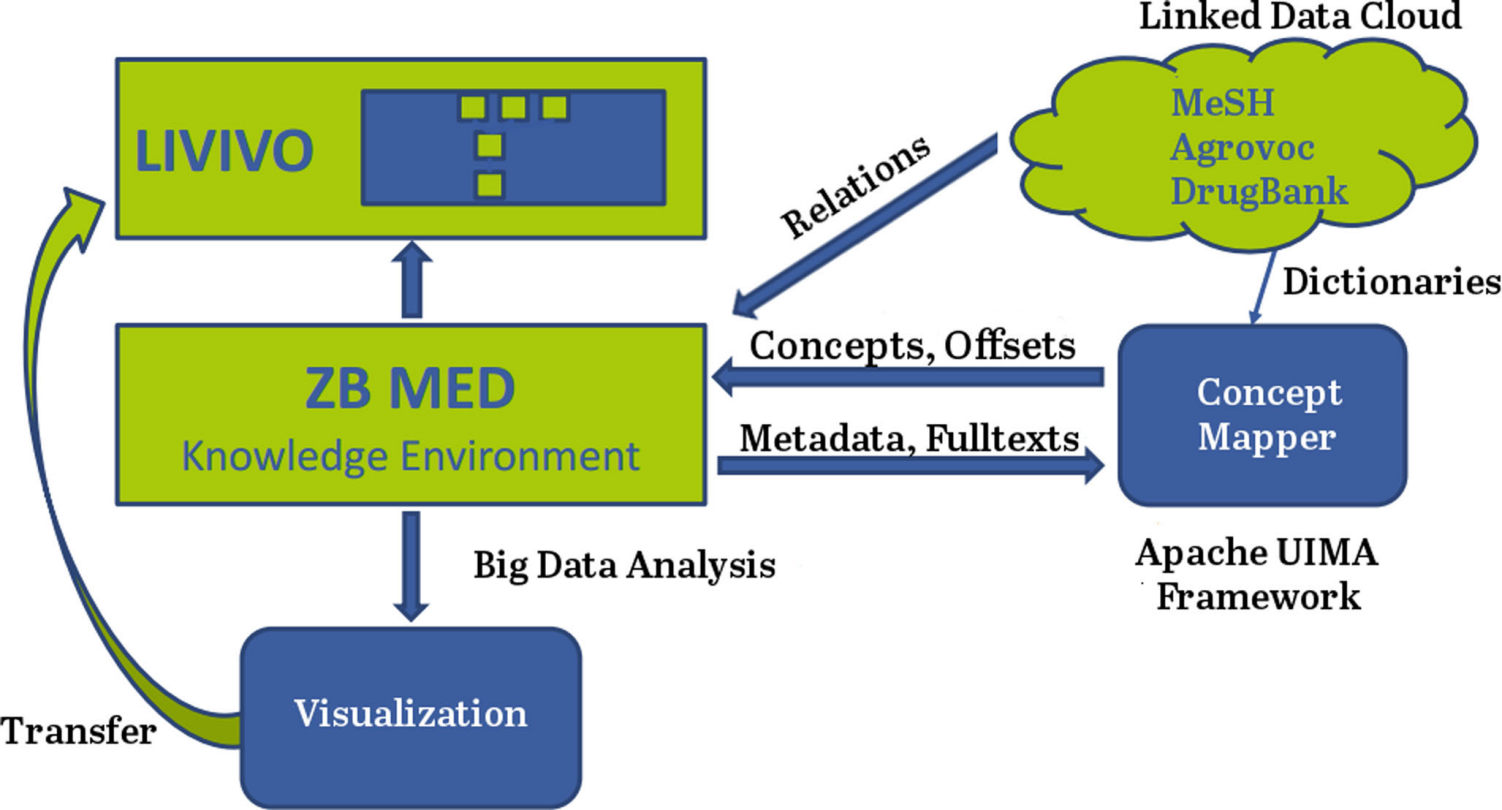
The ZB MED Knowledge Environment (KE) built upon literature resources with meta-data and fulltexts that are annotated using a UIMA-based text and data mining workflow. Dictionaries with concepts as well as relations of concepts are derived from MeSH, AGROVOC, and DrugBank. Big data analysis is conducted on the ZB MED KE that is transferred into LIVIVO as potentially novel visualization techniques

In order to realize the One Health paradigm, it is necessary to connect and interlink interdisciplinary knowledge domains by resolving the heterogeneous properties of data and information in databases and literature resources. In the domain of life sciences, the different knowledge layers have to be modeled by different entities, processes, and relationships for achieving an integrated knowledge environment [5]. Entities and their interactions are represented by processes with their relationships. The representation requires different views that depend on the respective domain. The domain context of pharmaceutical compounds interacting with patient groups needs a different environment for representing the knowledge than cellular processes of interacting cellular components. Hence, the ZB MED Knowledge Environment (ZB MED KE) is introduced in order to provide a general and persistent data layer for being able to model, store, and link semantic concepts within literature resources and databases of the life science domain. The ZB MED KE enables data curation, data enrichment, quality assurance, and export into the LIVIVO search database. The data enrichment of the literature resources is conducted with the annotation of life science concepts as standoff annotations in document texts. The semantic enrichment is prototypically implemented using a UIMA^14^ -based text and data mining workflow which also preserves the relational properties of the semantic concepts using a Neo4j^15^-based graph database [8]. The ZB MED KE is visualized in Fig. 2. There are several stages of expansion for the ZB MED KE involving natural language processing and ontology mapping.

### Mapping of Life Science Ontologies

In the text and data mining workflow employed for the semantic enrichment of the ZB MED KE, there are used different life science knowledge resource, namely *DrugBank* [7], *MeSH* [13], and *AGROVOC* [2]. *DrugBank* is a comprehensive resource for pharmaceutical compounds that offers the classification of approved drugs according to the *Anatomical Therapeutic Classification of Drugs and Drug Dosages* (ATC) that is offered and maintained by the *World Health Organization*. The *Medical Subject Headings* (MeSH) are maintained by the *National Library of Medicine* providing a hierarchy of relevant concepts to the medical domain. The *Multilingual agricultural thesaurus AGROVOC* is maintained by the *Food and Agricultural Organization of the United Nations* (FAO) that offers an ontological representation of agricultural and agriculture related concepts. All three resources are available as linked data. Potentially. There is an overlap with other existing knowledge bases, for example because of the intersection of the modeled domain with generic views or certain processes. In literature retrieval, the specificity of a knowledge resource for a specific domain is getting blurred by the overlay of generic concepts. There, specific concepts that represent the highest granularity of retrieved information are perturbed because of the high frequency of generic concepts [9]. Therefore, a mapping amongst the various knowledge resources has to be achieved in order to link common and generic concepts for the purpose of emphasizing highly specific concepts for the respective knowledge domain. This is required to improve the user experience in literature retrieval by providing a better resolution of semantic concepts that are used for query expansion, aggregation of search results, and concept based ranking methods. The standard scoring method is the fraction of term frequency and inverse document frequency (TF-IDF). TF-IDF is used for normalizing search results of matching terms according to the frequency of the terms in the complete corpus. In case of domain specific concepts, similar ranking function are derived as fraction of the concept frequency and the inverse document frequency (CF-IDF) [4]. The CF-IDF poses novel challenges compared to TF-IDF because concepts usually comprise spelling variants, synonyms, or even abbreviations because they have a large impact on the accuracy of the relevancy ranking.

### Natural Language Processing in LIVIVO

Relevancy ranking with CF-IDF has a high demand of accuracy for the Natural Language Processing methodology when identifying spelling variants, synonyms, and abbreviations of domain specific concepts. Therefore, current work on LIVIVO focuses on the development of linguistic approaches for named entity recognition and named entity disambiguation. These approaches are highly dependent on the syntax, the semantics, and the life science context. Ambiguous abbreviations and acronyms are frequently used in the life sciences [14]. Methodologies like Part-of-Speech (POS) tagging can support the correct identification in the process of natural language processing.

Furthermore, LIVIVO needs to deal with the detection of redundant content. Redundant content occurs, as LIVIVO compiles a variety of data sources which contain in part reciprocally or even intrinsically redundant content. Redundant content occurs when the same publication has multiple records in the literature resources. From the user‘s perspective, these occurrences should not be regarded as different citations. Redundancy may emerge in the LIVIVO data base at different levels that requires filtering in multiple steps. The identification of redundancies will include the disambiguation of document identifiers, type, and authors.

Another challenge in the area of natural language processing will have to deal with multilingualism in the corpus of LIVIVO as a multilingual search engine. Natural language processing is required for translating search queries into the respective document languages. Search terms have to be translated into concepts of multilingual thesauri such as AGROVOC that has translations in about 20 languages or MeSH that has translations into English, German, Chinese, and other languages.

## Conclusion and Outlook

LIVIVO takes on the required task of vertically integrating information from divergent research areas in the life sciences. The described ZB MED KE provides a core linking heterogeneous information from structured and unstructured sources. Future work will include expanding the range of ontologies employed in order to generate user friendly visualisations and functions for fine-tuning and stepwise filtering of query results. In addition including more research data and non-English documents in the data base are considered as unique features in comparison to other data bases such as Google Scholar or PubMed. Ultimately the semantic and vertical search capabilities of LIVIVO will allow answering complex questions and even generating new research questions in the life sciences.

## Footnotes

1
https://www.livivo.de [Accessed October 2016]

2
https://www.ncbi.nlm.nih.gov/pubmed [Accessed October 2016]

3
https://www.ebscohost.com/academic/agricola [Accessed October 2016]

4
https://www.ncbi.nlm.nih.gov/nlmcatalog [Accessed October 2016]

5
http://www.egms.de/dynamic/en/index.htm [Accessed October 2016]

6
https://www.loc.gov/marc/bibliographic/ [Accessed October 2016]

7
http://www.dnb.de/EN/Standardisierung/Formate/MAB/mab_node.html [Accessed October 2016]

8
https://www.ncbi.nlm.nih.gov/pubmed [Accessed October 2016]

9
http://dublincore.org/ [Accessed October 2016]

10
https://lucene.apache.org/solr/ [Accessed October 2016]

11
http://endnote.com/ [Accessed October 2016]

12
https://www.refworks.com [Accessed October 2016]

13
https://www.citavi.com/ [Accessed October 2016]

14
https://uima.apache.org/ [Accessed October 2016]

15
https://neo4j.com/ [Accessed October 2016]
